# The Molecular Epidemiological Study of HCV Subtypes among Intravenous Drug Users and Non-Injection Drug Users in China

**DOI:** 10.1371/journal.pone.0140263

**Published:** 2015-10-14

**Authors:** Jun Tao, Jun Liang, Hui Zhang, Lijian Pei, Han-zhu Qian, Matthew C. Chambers, Yan Jiang, Yao Xiao

**Affiliations:** 1 Vanderbilt Institution for Global Health, Vanderbilt University, Nashville, Tennessee, United States of America; 2 National AIDS Reference Laboratory (NARL), National Center for AIDS/STD Control and Prevention (NCAIDS), Chinese Center for Disease Control and Prevention (China CDC), Beijing, China; 3 Department of Medicine, Vanderbilt University, Nashville, Tennessee, United States of America; 4 Department of Biomedical Informatics, Vanderbilt University, Nashville, Tennessee, United States of America; University of Cincinnati College of Medicine, UNITED STATES

## Abstract

**Background:**

More than half of intravenous drug users (IDUs) in China suffer from the Hepatitis C virus (HCV). The virus is also more prevalent in non-injection drug users (NIDUs) than in the general population. However, not much is known about HCV subtype distribution in these populations.

**Methods:**

Our research team conducted a cross-sectional study in four provinces in China. We sampled 825 IDUs and 244 NIDUs (1162 total), genotyped each DU’s virus, and performed a phylogenetic analysis to differentiate HCV subtypes.

**Results:**

Nucleic acid testing (NAT) determined that 82% percent (952/1162) of samples were HCV positive; we subtyped 90% (859/952) of these. We found multiple HCV subtypes: 3b (249, 29.0%), 3a (225, 26.2%), 6a (156, 18.2%), 1b (137, 15.9%), 6n (50, 5.9%), 1a (27, 3.1%), and 2a (15, 1.7%). An analysis of subtype distributions adjusted for province found statistically significant differences between HCV subtypes in IDUs and NIDUs.

**Discussion:**

HCV subtypes 3b, 3a, 6a, and 1b were the most common in our study, together accounting for 89% of infections. The subtype distribution differences we found between IDUs and NIDUs suggested that sharing syringes was not the most likely pathway for HCV transmission in NIDUs. However, further studies are needed to elucidate how NIDUs were infected.

## Introduction

A meta-analysis of studies from 25 countries estimated that 60–80% of intravenous drug users (IDUs) were infected with the Hepatitis C virus (HCV). [1] In China, HCV prevalence ranged from 50% to 95% among IDUs; [2–4] the estimated incidence rate was from 11.6 to 29 per 100 person-years. [5–7] About 1.6 million IDUs tested positive for HCV-antibodies in 2011. [1] The HCV epidemic in non-injection drug users (NIDUs) is not well studied, despite a meta-analysis that reported an HCV prevalence of 2.3–35.3%. [8] Chronic HCV infection was strongly associated with liver cirrhosis and hepatocellular carcinoma (HCC); 5–20% of HCV chronic patients progress to liver cirrhosis in 20 to 25 years, and around 4% will suffer from HCC. [9,10] Sharing needles, syringes, and drug-preparation equipment has been demonstrated as the most common HCV transmission pathway in IDUs. [11] Sexual HCV transmission was previously thought to be unlikely, but recent research has reported a higher likelihood. [12–14]

HCV treatment can be effective in reducing risk for HCV-related liver diseases and HCV secondary transmission. [15] However, patients infected by different HCV genotypes do not respond to treatment in the same way. Compared to HCV genotypes 2, 3, 5 and 6, types 1 and 4 are treated with a higher dose of medication taken for a longer period but are still less likely to achieve sustained virologic response (SVR). [16–19] SVR is non-detection of HCV RNA at the end of the treatment and for the following six months. Recent research has suggested treatment efficacy also differs at subtype level. [20–22] However, few studies have focused on these treatment efficacy differences in the Chinese population. Furthermore, little is known about HCV subtype distribution among DUs, especially NIDUs. Hence, studies are needed to fill in this knowledge gap and further explore the association between HCV subtype and treatment efficacy.

## Methods

### Study population

To describe HCV subtype distribution among DUs in China, we conducted a cross-sectional study in four provinces. Between 2008 and 2011, we sampled 1,162 HCV-infected DUs from the national networks of methadone clinics and the HIV sentinel surveillance clinics in the Chinese provinces of Guizhou, Hebei, Jiangsu, and Shanxi. Two rounds of HCV-antibody enzyme-linked immunsorbent assays (ELISA) were used to determine HCV serostatus. All our study participants tested positive in both rounds. The national clinics de-identified participants’ demographic data and sent both the data and their blood samples to the Center for Disease Control (CDC) in China. The de-identified dataset still included demographic and behavioral information. Our study was approved by the Vanderbilt University School of Medicine IRB (number: 131253).

### HCV RNA extraction and nucleic acid testing (NAT)

At the CDC, we used participants’ blood samples to determine their HCV subtypes. We extracted HCV RNA using the MagNA Pure LC automated platform (*Roche Ltd*, *Basel*, *Switzerland*). The nucleic acid test (NAT) to detect the virus used a nested reverse transcription polymerase chain reaction (RT-PCR) process. First, we amplified a 170bp fragment in the 5’-untranslated region (5’-UTR). The primers we used for this process have been previously published (see S1 Text). [23] Secondly, we ran a one-step RT-PCR using TransScript^TM^ One-Step RT-PCR Supermix *(Beijing TransGen Biotech CO*. *LTD*, *China*) as the reagent mixture. It contained 5μL RNA extraction, 17μL DEPC water, 25μL Mix (dNTP, MgCl_2_ and buffer), 1μL mixture of enzymes (MMLV reverse transcriptase, Taq DNA polymerase and RNase Inhibitor), 1μL sense primer (5’-UTR-IS), and 1μL antisense primer (5’-UTR-IAS). We ran this first RT-PCR round for 30 min. at 50°C, 2 min. at 94°C, followed by 30 cycles of 30 sec. at 94°C, 30 sec. at 55°C, and 1 min. at 72°C. We allowed 7 min. at 72°C for elongation, and then stored the PCR product at 4°C. Third, we ran another nested-PCR step using 2×Taq Master Mix *(Tiangen Biotech CO*. *LTD*, *Beijing*, *China*) as the reaction mixture. It contained 2μL PCR products from RT-PCR, 21μL DEPC water, 25μL Mix (Taq DNA polymerase, dNTP, loading buffer, MgCl_2,_ and buffer), 1μL sense primer (5’-UTR-NS), and 1μL antisense primer (5’-UTR-NAS). We ran this round for 2 min. at 94°C, followed by 34 cycles of 30 sec. at 94°C, 30 sec. at 55°C, and 1 min. at 72°C. We used the same elongation and storage protocol as we did in the first step.

We amplified genes using a GeneAmp PCR 9700 thermal cycler (Applied Biosystems, NY, USA), and then used gel electrophoresis to detect the target DNA fragments.

### Amplification of the sequencing fragment in HCV NS5B region

For the RNA samples that tested positive for HCV, we amplified a 350bp fragment in the NS5B region. We then applied a nested RT-PCR procedure similar to the NAT procedure, except using different primers (S1 Table). We used gel electrophoresis to detect the target fragment. Amplified products were sent to Biomed Laboratory (Beijing, China) for sequencing.

### Subtyping and statistical analysis

We used ChromasPro 1.5 to trim and assemble sequences. Then we used BioEdit 4.0 to perform multiple alignments against the NS5B reference sequences from the HCV Sequence Database (*https*:*//hcv*.*lanl*.*gov/content/sequence/HCV/ToolsOutline*.*html*). Finally, we ran Mega 4.0 to conduct the phylogenetic analyses. Subtype information was read from the resulting phylogenetic trees (using the neighbor-joining method with the Kimura-2-parameter).

We compared HCV subtype distributions among demographic variables using a chi-squared test. To control for confounding effects by region, we analyzed the subgroups on a per-province basis. We conducted all analyses using Statistical Analysis System 9.3 (*SAS Institute Inc*, *Cary*, *NC*, *USA*).

## Results

### Demographic characteristics

The median age in our study population was 38 years (interquartile range [IQR]: 32, 43). Most participants were of Han ethnicity, male, single, and had less than an 8^th^-grade education. Our sample population was not spread equally across each province: 26.6% were from Guizhou, 27.1% from Shanxi, 30.2% from Jiangsu, and 16.1% from Hebei. Nearly equal numbers of participants were sampled from methadone clinics and HIV sentinels (560 vs. 602). Most participants in our study population (77.2%) were IDUs.

### Results of NAT and subtyping

**Fig 1:**
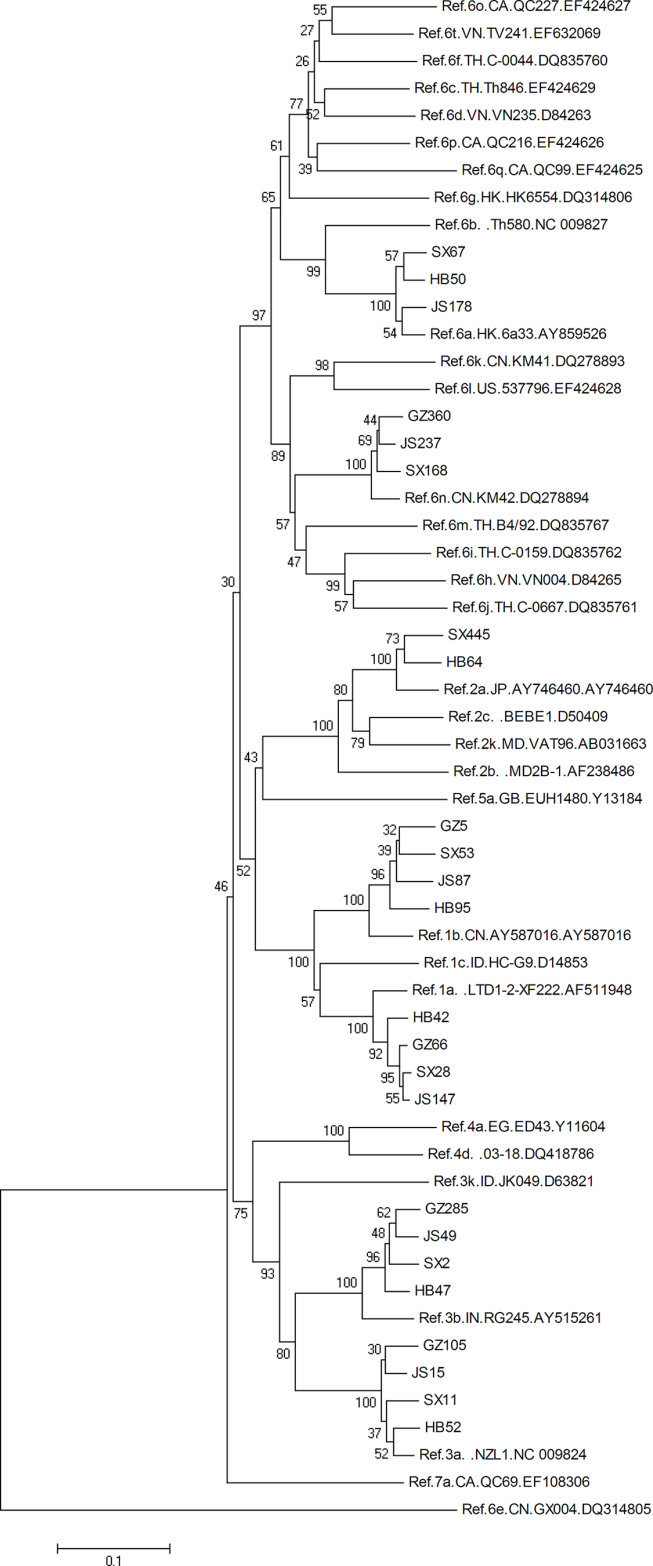
The phylogenetic tree constructed with sequences from HCV NS5B region among Chinese drug users.

In this study, 952 (81.9%) DUs tested positive for HCV RNA, and we successfully subtyped 859 of those 952 (90.2%). The proportion of NAT positive and subtyping rate differed by province and sample source (Table 1). For example, participants from Hebei were less likely to be NAT positive and to be subtyped. Participants from methadone clinics also had a low rate of RNA detection and subtyping. The result of our phylogenetic analysis on the HCV subtypes can be seen in Fig 1.

**Table 1:** Description of demographic and behavioral characteristics among HCV-infected drug users in China.

Covariates	No. of HCV infected (N = 1,162)	No. of NAT positive (N = 952)	No. of subtyped (N = 859)
Median Age (year, IQR)	38 (32, 43)	38 (32, 43)	38 (32, 43)
Gender			
Male	906 (78.0)	750 (78.8)	676 (78.7)
Female	256 (22.0)	202 (21.2)	183 (21.3)
Ethnicity			
Han	1,112 (95.7)	915 (96.1)	829 (96.5)
Other	50 (4.3)	37 (3.9)	30 (3.5)
Province			
Guizhou	309 (26.6)	258 (27.1)	246 (28.6)
Shanxi	315 (27.1)	256 (26.9)	221 (25.7)
Jiangsu	351 (30.2)	292 (30.7)	277 (32.3)
Hebei	187 (16.1)	146 (15.3)	115 (13.4)
Education years			
Over 8	129 (11.6)	105 (11.5)	98 (11.9)
6–8	641 (57.6)	528 (57.8)	477 (57.9)
0–5	342 (30.7)	280 (30.7)	249 (30.2)
Marriage			
Married	410 (36.0)	350 (37.5)	315 (37.4)
Single	597 (52.4)	470 (50.4)	421 (49.9)
Divorced	133 (11.6)	113 (12.1)	107 (12.7)
Missing	22	19	16
Sample source			
Methadone clinics	560 (48.2)	460 (48.3)	393 (45.8)
HIV Sentinels	602 (51.8)	492 (51.7)	466 (54.2)
Method of drug use			
Injection	825 (77.2)	672 (71.1)	601 (75.5)
Non-injection	244 (22.8)	211 (23.9)	195 (24.5)
Missing	93	79	63

IQR: interquartile range; NAT: nucleic acid testing.

### The distribution of HCV subtypes

**Fig 2:**
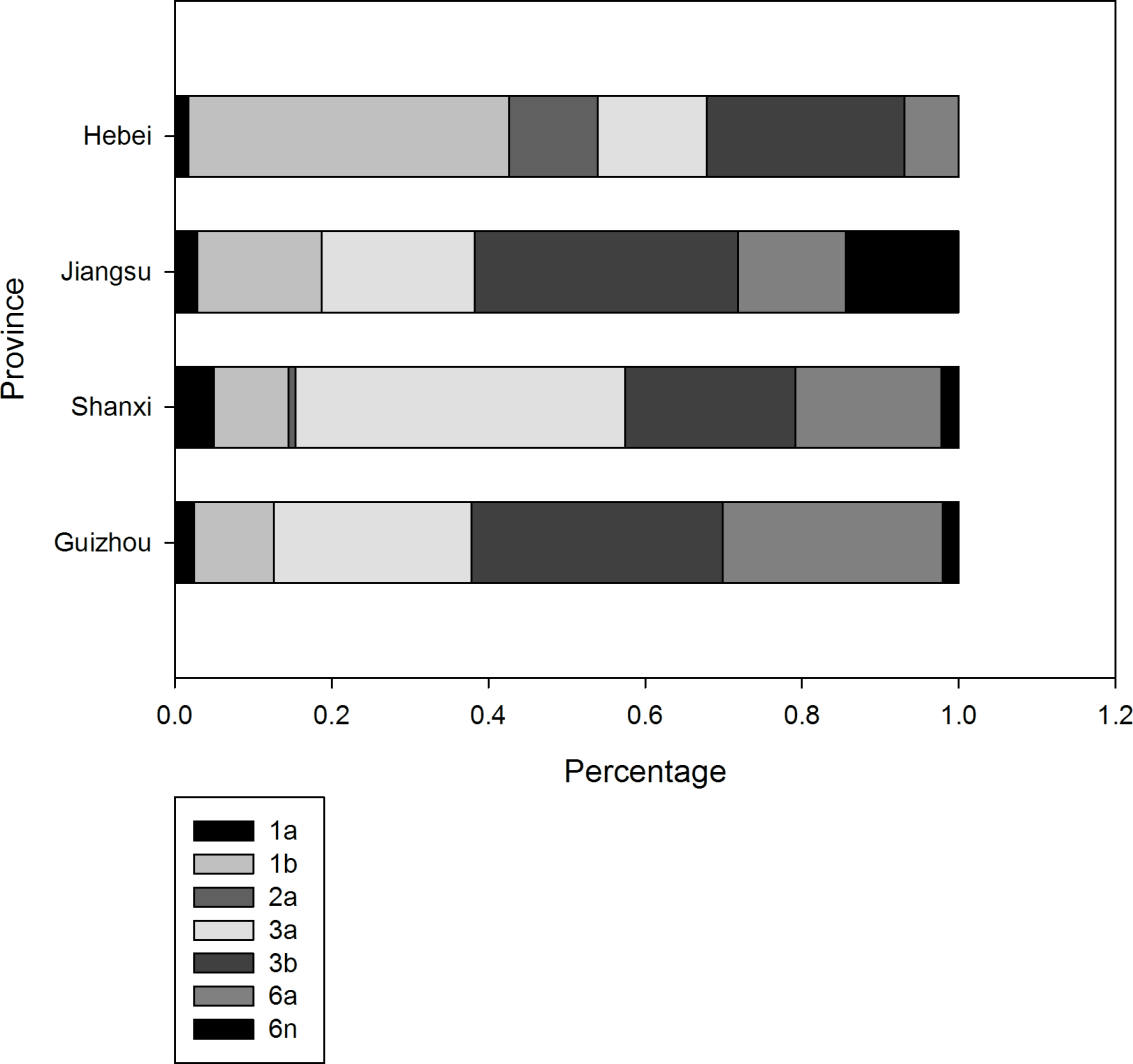
The distribution of HCV subtypes among Chinese drug users in four provinces.

Table 2 describes the distribution of subtypes among HCV-infected DUs. HCV 3b (249, 29.0%), 3a (225, 26.2%), and 6a (156, 18.2%) were the most common strains among drug users. Bivariate analysis suggested that frequencies of HCV subtype differed between IDUs and NIDUs (Table 2). They also differed by province (Fig 2). When we stratified the data by province, the difference in frequencies of HCV subtypes between IDUs and NIDUs was statistically significant.

**Table 2:** HCV subtype distribution between intravenous drug users (IDUs) and non-injection drug users (NIDUs) in China.

	HCV Subtypes (%)							
DUs	1a	1b	2a	3a	3b	6a	6n	Total
IDUs	20 (3.3)	92 (15.3)	12 (2.0)	175 (29.1)	173 (28.8)	92 (15.3)	37 (6.2)	601
NIDUs	6 (3.1)	41 (21.0)	3 (1.5)	33 (16.9)	46 (23.7)	55 (28.2)	11 (5.6)	195
Missing	1 (1.6)	4 (6.4)	0 (0.0)	17 (27.0)	30 (47.6)	9 (14.2)	2 (3.2)	63
Total	27 (3.0)	137 (16.0)	15 (1.8)	225 (26.2)	249 (29.0)	156 (18.2)	50 (5.8)	859

## Discussion

Our study population was on average older than those in other studies, but the ratio of males to females was similar (3.5:1). [24,25] Previous studies have reported that young DUs are more likely to inject drugs while older DUs may switch to non-injection drugs. [15] Since our study population included both IDUs and NIDUs, it was not surprising that our participants were older than the general IDU populations in the literature. Older DUs were more likely to be married and sexually active. Thus, they should have a higher chance of transmitting HCV to their sexual partners than younger DUs would.

The main HCV subtypes in our DUs were 3b, 3a, 6a, and 1b, which was concordant with other studies. [24,26] By contrast, in Korea, the main strains among DUs were 1b and 2a/2c [27], while in Vietnam, 1a, 1b, and 6a were the most common. [28] Because we sampled HCV-infected DUs from 4 provinces, our study population better represented Chinese DUs than previously published studies, which focused on small samples of IDUs in a single geographic region. We replicated the geographic distribution of HCV subtype found by a previous study [29]: 1b and 3b were the main strains in the northern part, 3a and 3b in the central part, and 3b and 6a in the southern part. However, HCV subtypes, which used to be seen only in a certain geographic region, were also found in different regions. Migration may have broken the geographic limitation of HCV subtypes.

We found different HCV subtype distributions between IDUs and NIDUs, which contradicted research conducted in the Netherlands. [30] Van den Berg *et al*. found frequencies of HCV subtypes to be similar between IDUs and NIDUs. In our study, HCV subtype 1b, 3b, and 6a were the main strains in NIDUs, whereas 3a and 3b were the most common in IDUs. Our finding was biologically plausible because certain HCV subtypes are mainly transmitted by IDUs through sharing of needles, while true NIDUs must be infected via a different pathway, such as sexual transmission. We say “true NIDUs” because we observed that a small proportion of HCV sequences from IDUs and NIDUs clustered into the same group in our phylogenetic analyses (Fig 1). This indicated that some NIDUs injected drugs but reported as non-injection DUs. However, response bias alone cannot explain the HCV epidemic among NIDUs, and more research is needed to understand the mechanism of HCV transmission in this population.

This study had several limitations. The quality of plasma is critical for RNA extraction and genotyping. Variable plasma quality from clinics in different provinces can be observed, and the HCV genotyping failures might be largely dependent on that variable. Thus, we consider HCV subtype to be missing at random (MAR). Drug use method (IDU vs. NIDU) may also be MAR, as the majority of missing responses were from participants from Guizhou. As a result, when conducting analyses between provinces, our findings could be either inflated or attenuated. As mentioned before, response bias from DUs seems to have caused some of them to be misclassified. However, we partially offset this by studying a large sample covering four geographic areas in China. We successfully replicated the geographic distribution of HCV subtype from previous studies, and discovered that the HCV subtype distribution was different between IDUs and NIDUs. Our study provided information for future HCV transmission research among NIDUs, and was potentially useful for guiding HCV treatment in China.

## Supporting Information

S1 Table Table 1 Primers for the amplification of target fragments in HCV 5’-UTR and NS5B. (DOCX)

S1 Text related published paper in a Chinese Journal. (PDF)
