# Supplementary material for: The Molecular Epidemiological Study of HCV Subtypes among Intravenous Drug Users and Non-Injection Drug Users in China
Source: PLoS One. 2015 Oct 14;10(10):e0140263. doi: 10.1371/journal.pone.0140263 (PMC4605846; doi:10.1371/journal.pone.0140263)
Supplement: S1 Table — (DOCX) [file pone.0140263.s001.docx]

Table 1 Primers for the amplification of target fragments in HCV 5’-UTR and NS5B

| Primers | Sequence (5’-3’) | Location |
| --- | --- | --- |
| 5'-UTR-IS^*^ | GCCATGGCGTTAGTAYGAGT | 82~101 |
| 5'-UTR-IAS^†^ | TTTCGCRACCCAACRCTACT | 276~257 |
| 5'-UTR-NS^‡^ | AGTGTCGTRCAGCCTCCAGG | 99~118 |
| 5'-UTR-NAS^§^ | ACCCAACRCTACTCGGCTAG | 269~250 |
| NS5B-IS | CCA ATH SMC ACT ACC ATC ATG GC | 8001~8020 |
| NS5B-IAS | TGG AGT GTG NCK RGC HGT YTC C | 8792~8810 |
| NS5B-NS | CGT ATG AYA CCM GVT GYT TTG A | 8257~8275 |
| NS5B-NAS | CCT RGT CAT AGC HTC CGT GAA | 8616~8633 |

* sense primer for the initial PCR; † anti-sense primer for the initial PCR;

‡ sense primer for the nested PCR; § anti-sense primer for the nested PCR;
