# Supplementary material for: The Molecular Epidemiological Study of HCV Subtypes among Intravenous Drug Users and Non-Injection Drug Users in China
Source: PLoS One. 2015 Oct 14;10(10):e0140263. doi: 10.1371/journal.pone.0140263 (PMC4605846; doi:10.1371/journal.pone.0140263)
Supplement: S1 Text — (PDF) [file pone.0140263.s002.pdf]

·论 著·

贵州吸毒人群丙型肝炎病毒感染者的基因分型研究

陶钧[1]，刘勇[2]，童毅[2]，于石成[3]，张辉[1]，蒋岩[1]，肖瑶[1]

1. 中国疾病预防控制中心性病艾滋病中心参比室，北京 102206；
2. 贵州省疾病预防控制中心艾滋病防治所，贵州贵阳 550004
3. 中国疾病预防控制中心卫生统计教研室，北京 102206

摘要：目的 了解贵州吸毒人群丙型肝炎病毒（HCV）感染者的基因亚型分布，为该省吸毒人群HCV临床治疗和估计HCV造成疾病负担提供参考信息。**方法** 抽取贵州省部分美沙酮门诊和强制戒毒所吸毒人员血浆样本，经过初筛和复检，筛选HCV抗体阳性样本；提取HCV核酸后，用巢式RT-PCR扩增5′-UTR区段进行HCV核酸的定性检测（nucleic acids technology，NAT），以筛选HCV核酸阳性样本；再用巢式RT-PCR扩增NS5B区段，对凝胶电泳条带阳性扩增产物进行测序，将清理后的序列与HCV Database提供的NS5B区段参考序列比对，使用MEGA 4.0做系统进化树，进行基因分型。**结果** 309份抗体阳性样本中核酸阳性258份（83.5%），成功分型246份，10份测序区扩增失败，2份测序失败。246份中1a 6份，1b 25份，3a 62份，3b 79份，6a 69份，6n 5份。吸毒人群HCV感染者的基因亚型仅在不同性别和吸毒方式间的分布上差异有统计学意义。NAT（－）与NAT（＋）样本HCV抗体酶联免疫吸附法检测S/CO比值差异无统计学意义。**结论** 贵州吸毒人群HCV感染者的基因型主要为3型和6型、少数为1型，未发现2型、4型和5型；性别和吸毒方式影响吸毒人群HCV感染者基因亚型的分布。

关键词： 吸毒人群；HCV感染者；基因分型

中图分类号： R373.2 **文献标识码：** A **文章编号：** 2095-0136（2012）02-0117-06

Hepatitis C virus genotyping analysis among drug users in Guizhou, China

TAO Jun*，LIU Yong，TONG Yi，YU Shi-cheng，ZHANG Hui，JIANG Yan，XIAO Yao

** National AIDS/HCV Reference Laboratory，National Center for AIDS/STD Control and Prevention，Chinese Center for Disease Control and Prevention，Beijing 102206，China*

Corresponding authors：JIANG Yan，E-mail：jiangyan03@263.net；XIAO Yao，E-mail：xiaoyao@chinaaids.cn

Abstract： **Objective** To study the distribution of hepatitis C virus（HCV）genotypes among drug users in Guizhou，China and provide guidance for HCV clinical treatment and estimate the burden of hepatitis C infection in this special population. **Methods** Serum samples were collected from drug users in Methadone Clinics and Detention Centers in Guizhou，China. ELISA-screened anti-HCV positive samples were further subject to HCV RNA extraction using Nucleic Acids Technology（NAT）that targets the 5′ untranslated region（5′-UTR). Next，the NS5B region was amplified for sequencing analysis，and software ChromasPro，BioEdit and MEGA 4.0 were used to construct the phylogenetic tree. **Results** There was no significant difference in the S/CO ratios between ELISA and NAT detection. More male drug users were found than females；younger age and divorce were additional risk factors in drug addiction. 258 samples showed HCV RNA positive out of a total of 309 anti-HCV positive ones；successful sequencing was done with 246 samples for further genotyping analysis. Genotypes 1a，1b，3a，3b，6a and 6n were identified in 6，25，62，79，69 and 5 samples，respectively. **Conclusions** Genotype 3 and 6 are the main HCV strains among drug users in Guizhou，China.

Key words： Drug user；HCV；Genotype

基金项目： 2011年国家丙型肝炎防治经费项目

作者简介： 陶钧，硕士研究生，主要从事HCV基因亚型研究

通讯作者： 蒋岩，E-mail：jiangyan03@263.net；肖瑶，E-mail：xiaoyao@chinaaids.cn

© 1994-2012 China Academic Journal Electronic Publishing House. All rights reserved. http://www.cnki.net

丙型肝炎病毒（HCV）的传播主要为医源性和不安全注射，性传播效率较低。在不安全注射中，静脉注射吸毒人群共用注射器是造成该人群 HCV 感染率高达 60%～80%的主要原因。HCV 感染人群中大约 30%可以自然清除 HCV，70%～80%进展为慢性感染[1]，其中约 30%的慢性感染者会出现慢性肝脏疾病，例如肝纤维化、肝硬化和原发性肝细胞癌[2-3]。目前，HCV 的临床治疗药物主要有干扰素（IFN）-α 和利巴韦林[4]，而个体对于药物治疗的敏感性与宿主因素[5]、感染的 HCV 基因型[4]及基线病毒载量相关[6-7]。其中，HCV 基因亚型是预测临床转归最重要的一个指标[8]。持续病毒学应答（sustained virological response，SVR，指停药 6 个月后，核酸检测仍为阴性）是评价治疗是否有效的重要指标。1 型和 4 型 HCV 感染者经过 12 个月大剂量 IFN-α 和利巴韦林联合治疗，仅有 40%～50%患者出现 SVR；而 2 型和 3 型 HCV 感染者经过 6 个月的治疗，超过 70%～80%的患者出现 SVR，5 型和 6 型 HCV 感染者对于治疗的敏感性类似于 2 型和 3 型[9-11]。因而了解吸毒人群 HCV 感染者的基因亚型分布，可以为临床治疗和估计 HCV 造成的疾病负担提供信息和指导。

1 对象与方法

1.1 对象 研究对象来自贵州省 3 个美沙酮门诊和 2 个强制戒毒所的吸毒人群，经酶联免疫吸附方法初筛和复检共获取 309 份 HCV 抗体阳性样本。其中，170 份来自美沙酮门诊，139 份来自强制戒毒所。收集的研究对象背景信息包括：性别、年龄、民族、职业、婚姻状况、教育背景和吸毒方式。

1.2 方法

1.2.1 HCV 抗体筛查 使用万泰（WT）-丙型肝炎病毒抗体诊断试剂盒（酶联免疫吸附法，ELISA）对样本进行初筛，对于 S/CO 比值>0.8 的样本再用 Ortho-丙型肝炎病毒抗体诊断试剂盒（ELISA）进行复检。两次 ELISA 检测 S/CO 比值均>1 定义为 HCV 抗体阳性样本。

1.2.2 核酸提取 核酸提取使用 Roche 公司（瑞士）的 MagNA Pure LC 核酸自动提取仪，试剂（MagNA Pure LC Total Nucleic Acid Isolation Kit，Roche）和耗材（sample carriages，reagent tube，tips，tip stands，processing carriages，drop catcher，and waste bags）为 MagNA Pure LC 专用。200 μl 样本经过胍盐裂解和蛋白酶 K 消化后，核酸与磁珠结合。经过 3 次洗脱后，加热使磁珠与核酸分离，最终洗脱体积为 50 μl。

1.2.3 5′-UTR 区段扩增 巢式 RT-PCR 扩增 HCV 基因组高保守区段 5′-UTR，筛选 HCV 核酸阳性样本。一轮 RT-PCR 体系（TransScriptTM One-Step RT-PCR Supermix，全式金）为：5 μl 模板，17 μl DEPC 水，25 μl Mix（dNTP，$MgCl_2$，buffer 的混合物），1 μl 酶混合物（MMLV 逆转录酶、Taq 酶和 RNA 水解酶抑制剂），各 1 μl 的外侧上游引物和外侧下游引物。一轮 RT-PCR 反应条件为：经过 50℃ 30 min 逆转录和 94℃ 2 min 热启动后，PCR 循环 30 次（94℃ 30 s，55℃ 30 s，72℃ 1 min），延伸条件为 72℃ 7 min，4℃保存扩增产物；二轮 PCR 体系（2×Taq Master Mix，天根生化）：2 μl 模板，21 μl DEPC 水，25 μl Mix（Taq 酶、dNTP、loading buffer、$MgCl_2$ 和 buffer 的混合物），各 1 μl 的内侧上游引物和内侧下游引物。二轮 PCR 反应条件为：经 94℃ 2 min 热启动后，PCR 循环 34 次（94℃ 30 s，55℃ 30 s，72℃ 1 min），延伸条件为 72℃ 7 min，4℃保存扩增产物。使用的 PCR 仪为 ABI9700。引物序列和在 HCV 基因组中位置，见表 1。

1.2.4 NS5B 区段扩增 巢式 RT-PCR 扩增 HCV 基因组 NS5B 区段，以进行 HCV 基因分型。一轮 RT-PCR 和二轮 PCR 的反应体系及反应条件与 5′-UTR 区段扩增相同。引物序列和在 HCV 基因组中位置，见表 1。

表 1 实验使用引物序列

引物名称	序列（5′-3′）	基因组中的位置
5′-UTR-NZ	AGTGTCGTRCAGCCTCCAGG	99～118
5′-UTR-NF	ACCCAACRCTACTCGGCTAG	269～250
5′-UTR-WZ	GCCATGGCGTTAGTAYGAGT	82～101
5′-UTR-WF	TTTCGCRACCCAACRCTACT	276～257
NS5B-NZ	CGT ATG AYA CCM GVT GYT TTG A	8257～8275
NS5B-NF	CCT RGT CAT AGC HTC CGT GAA	8616～8633
NS5BWZ	CCA ATH SMC ACT ACC ATC ATG GC	8001～8020
NS5BWF	TGG AGT GTG NCK RGC HGT YTC C	8792～8810

注：NZ：内侧正向引物，NF：内侧反向引物；WZ：外侧正向引物，WF：外侧反向引物。

© 1994-2012 China Academic Journal Electronic Publishing House. All rights reserved. http://www.cnki.net

1. 2. 5　凝胶电泳及基因测序　配置 1％的琼脂糖凝胶，使用 Gold View Ⅱ 核酸染料和 2×10^3 bp marker。凝胶电泳电压为 150 V，时间为 30 min；在凝胶成像仪下观察结果，5′-UTR 区段阳性为核酸阳性样本，NS5B 区段阳性扩增产物送至博迈德基因测序公司测序。

1. 3　基因序列分析方法　用 ChromoPro1. 5 软件进行序列清理和拼接，用 Bio-Edit 4. 0 软件将清理拼接后的序列与 Los Alamos HCV database（http：//hcv. lanl. gov）提供 NS5B 区段参考序列比对；运用 MEGA 4. 0 做系统进化树，进行基因分型。

1. 4　统计学处理　利用 Microsoft Excel 2007 建立数据库，将年龄作为分组变量，即 15～25 岁，25～35 岁，35～45 岁，＞45 岁。将整理核对后的数据导入 SAS 9. 1 进行统计学分析。分析内容包括：样本来自人群的人口学和社会学信息描述；与静脉吸毒相关的多因素 logistic 回归；核酸定性检测（NAT）阳性与阴性样本抗体酶联免疫检测 S/CO 比值是否存在统计学差异；单因素分析 HCV 基因型和基因亚型分布在不同性别、年龄、民族、职业、婚姻状况、教育背景和吸毒方式间是否存在统计学差异。使用 OrginPro 8 作图。

2　结　果

2. 1　被检人群人口学信息　研究对象的平均年龄为 35 岁，男性 245 例，女性 64 例；汉族 287 例（92. 9％），回族和彝族等少数民族共 22 例（7. 1％）；无业/待业者 245 例（79. 3％），其他职业者及职业不详者 64 例（20. 7％）；未婚 158 例（51. 1％），已婚 74 例（23. 9％），离异/丧偶 37 例（12. 0％），婚姻状态不详 40 例（12. 9％）；教育程度以初高中为主（203 例，65. 7％），文盲和小学 51 例（16. 5％），大专及以上学历 18 例（5. 8％），教育程度不详 37 例（12. 0％）。

吸毒方式中，静脉注射吸毒者 158 例（51. 1％），单纯口吸或烫吸 71 例（23. 0％），吸毒方式不详 80 例（25. 9％）。多因素 logistic 回归分析显示（吸毒方式不详者未列入分析）：是否静脉注射吸毒与性别、年龄和婚姻状况差异有统计学意义（表 2）。男性静脉注射吸毒的比值比（odd ratios，*OR*）是女性的 2. 19 倍；低年龄组注射吸毒的风险高于高年龄组；未婚吸毒者静脉注射吸毒的比值比是已婚者 0. 31 倍，离异/丧偶和婚姻状况不详者静脉注射吸毒的风险均高于已婚者。

表 2　静脉注射吸毒危险因素的 logistic 回归分析

因素	例数	*OR* 值及 95％置信限（95％ *CI*）
性别		
女性*	55	1. 00
男性	174	2. 19（1. 09～4. 40）
年龄（岁）		
15～25	22	6. 86（1. 43～33. 01）
25～35	98	9. 10（3. 02～27. 46）
35～45	87	1. 97（0. 70～5. 60）
＞45*	22	1. 00
婚姻状况		
已婚*	64	1. 00
未婚	134	0. 31（0. 15～0. 63）
离异/丧偶	28	1. 74（0. 66～4. 57）
不详	3	1. 24（0. 07～20. 83）

注：* 表示参照组。

2. 2　酶联免疫检测结果　309 份 HCV 抗体阳性样本初筛 S/CO 比值中位数为 20. 47，最小值 1. 62，最大值 27. 30；复检 S/CO 比值中位数为 5. 30，最大值 5. 32，最小值 0. 48。图 1 显示：NAT 检测阴性与阳性样本的初筛 S/CO 比值分布相近，成组 *t* 检验结果：$t=0.01$，$P>0.05$，认为两者 S/CO 比值差异无统计学意义；图 2 为 NAT 检测阴性与阳性样本的复检 S/CO 比值分布，成组 *t* 检验结果：$t=1.33$，$P>0.05$，认为两者 S/CO 比值差异无统计学意义。

2. 3　HCV 核酸阳性及基因分型结果　309 份初筛复检 HCV 抗体均阳性样本中，核酸阳性样本数为

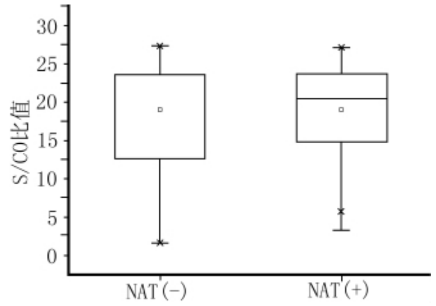

图 1　NAT（－）与 NAT（＋）ELISA 初筛 S/CO 比值

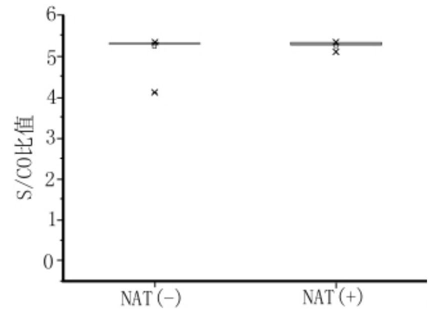

图 2　NAT（－）与 NAT（＋）ELISA 复检 S/CO 比值

© 1994-2012 China Academic Journal Electronic Publishing House. All rights reserved.　http://www.cnki.net

258 份（83.5%）。成功扩增 NS5B 区段并得到基因序列的样本数为 246 份，10 份测序区扩增失败，2 份测序失败。其中 1a 6 份（2.4%），1b 25 份（10.2%），3a 62 份（25.2%），3b 79 份（32.1%），6a 69 份（28.1%），6n 5 份（2.0%）；基因型主要为 3 型，其次为 6 型，1 型所占比例最少，未发现 2 型、4 型和 5 型。系统进化分析见图 3。

2.4　影响 HCV 基因亚型分布的因素　HCV 基因型和基因亚型分布与性别、年龄、民族、职业、婚姻状况、教育背景和吸毒方式的单因素分析结果显示：HCV 基因型和基因亚型分布仅在不同性别和不同吸毒方式间差异有统计学意义，χ^2 检验 P 值均 <0.05。在不同样本来源、年龄、民族、职业、婚姻状况和教育背景间的分布差异无统计学意义。表 3 显示：贵州吸毒人群中，男性 HCV 感染者的基因型主要为 3 型和 6 型，而 1 型、3 型和 6 型在女性 HCV 感染者中所占比例相近；静脉注射吸毒者和吸毒方式不详者的 HCV 基因型均以 3 型和

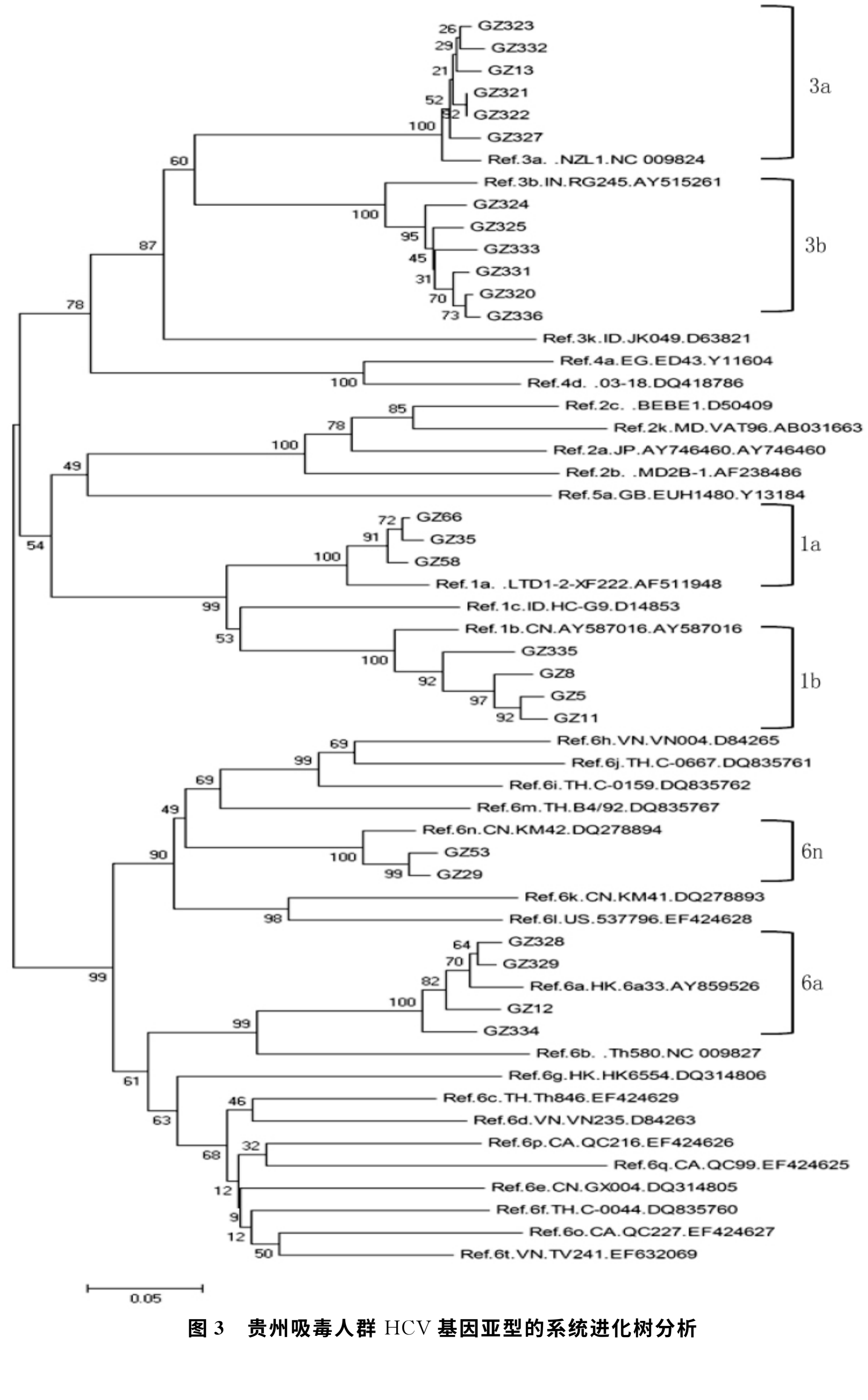

图 3　贵州吸毒人群 HCV 基因亚型的系统进化树分析

© 1994-2012 China Academic Journal Electronic Publishing House. All rights reserved.　http://www.cnki.net

表 3 HCV 基因亚型在不同性别和吸毒方式的吸毒人群间的分布情况

项目	例数	HCV 基因亚型					
		1a	1b	3a	3b	6a	6n
性别							
男性	197	4 (2.03)	14 (7.11)	52 (26.40)	69 (35.02)	53 (26.90)	5 (2.54)
女性	49	2 (4.08)	11 (22.45)	10 (20.41)	10 (20.41)	16 (32.65)	0
吸毒方式							
静脉注射	126	4 (3.17)	9 (7.14)	37 (29.37)	37 (29.37)	38 (30.16)	1 (0.79)
单纯口吸或烫吸	61	1 (1.64)	13 (21.31)	10 (16.39)	13 (21.31)	22 (36.07)	2 (3.28)
吸毒方式不详	59	1 (1.69)	3 (5.08)	15 (25.42)	29 (49.15)	9 (15.25)	2 (3.39)

6 型为主，而 1 型、3 型和 6 型在单纯口吸或烫吸的 HCV 感染者中所占比例也相近。

3 讨 论

吸毒人群以男性为主，年龄集中在 20～40 岁；大约 80%无业或待业，文化程度相对较低，未婚者超过 50%，静脉注射吸毒者占多数。男性、低年龄及已婚和离异/丧偶是静脉注射吸毒的危险因素。吸毒人群的流动性和多性伴使其成为 HCV 由高危人群向普通人群传播的桥梁人群。因而，需要了解该人群 HCV 感染基因型分布，以便合理治疗达到预防 HCV 二代传播的目的。

本研究显示：国产 HCV 抗体 ELISA 检测样本的 S/CO 比值区间范围大于进口试剂，主要是因为国产试剂 Cut Off 值较进口试剂低。因 HCV 感染者抗体滴度与 HCV 病毒载量间不存在相关性[12]，酶联免疫检测 S/CO 比值不能预测 NAT 检测结果，也不能反映 HCV 感染者的感染状况和评价 HCV 治疗效果。HCV 5′-UTR 区段高度保守，可作为检测核酸阳性的靶位区段，但因其高度保守，不同基因型在该区段的基因差异较小，不足以区分某些基因亚型[13]，且 6 型与 1b 的 5′-UTR 区段基因序列一致，会造成错误分型[11,14]。因此，多数研究认为包含基因亚型信息最为丰富的 NS5B 区段，适用于 HCV 基因亚型分布的流行病学研究[15-18]。本研究 HCV 核酸阳性样本占 HCV 抗体阳性的 85.3%，与报道的 HCV 感染后大约 30% 的自然清除率相符，但不排除某些个体因治疗而无法检测到核酸的情况。

贵州吸毒人群 HCV 感染者的基因亚型分布以 3 型为主，与国外报道 3 型主要在吸毒人群中传播一致[19]。其次是 6 型，因贵州处于中国南部，临近东南亚，而 6 型在东南亚分布较广[11,20]，所以该地区吸毒人群 HCV 感染 6 型的比例相对较高。在贵州吸毒人群中，1 型 HCV 感染者所占比例最少，未发现 2 型、4 型和 5 型。文献报道：1 型和 2 型主要集中在既往有偿献血者和血制品输入者中[21]。虽然本研究中未发现主要分布在中东地区的 HCV 4 型和在南非的 HCV 5 型，但广东等地已发现 HCV 4 型感染者[22]。便捷的交通和日益频繁的贸易合作打破 HCV 的地域性阻隔，从而导致了在中国的传播。70%～80%的 3 型和 6 型感染者经过短疗程低剂量的 IFN-α 和利巴韦林联合治疗可出现 SVR[9]。6 型对于联合治疗的敏感性与 3 型类似[10-11]。因而，在该人群中，开展 HCV 的早期治疗，可以有效降低二代传播，减少因 HCV 慢性感染而导致的肝脏继发病变。贵州吸毒人群中，HCV 感染者基因亚型在男性和女性间分布存在差异，国外亦有类似报道[23]。女性与单纯口吸或烫吸的 HCV 感染者基因亚型分布相似，而男性与静脉吸毒及吸毒方式不详的 HCV 感染者基因亚型分型相似。可能的原因是：女性单纯口吸或烫吸毒品的比例高于男性，而男性以静脉注射吸毒为主；单纯口吸或烫吸的 HCV 感染者可能经其他途径感染，而静脉注射吸毒的 HCV 感染者大部分因共用注射器而感染。但单纯口吸或烫吸与静脉注射吸毒的 HCV 感染者的基因序列有聚成一簇的现象，可能是吸毒者谎报吸毒方式[24]，也可能是与静脉注射吸毒的 HCV 感染者发生性行为而感染。

吸毒方式是影响吸毒人群 HCV 感染者基因亚型分布的主要行为学因素，但其他因素也可能影响 HCV 感染者的基因亚型分布。例如：吸毒方式在年龄组间、不同婚姻状况和不同教育背景间分布不同，但 HCV 基因型和基因亚型分布与以上 3 个因素均无关。以上现象也可能是因为存在混杂因素而造成统计学上无意义结果。本次研究中，民族对于吸毒人群 HCV 感染者基因亚型分布可能是假阴性结果，因为 93.5%的样本来自汉族；79%的吸毒人群为无业或待业状态，因而职业可能对于研究吸毒人群 HCV 感染者的基因亚型分布不是一个有价

© 1994-2012 China Academic Journal Electronic Publishing House. All rights reserved. http://www.cnki.net

值的影响因素。

本研究结果显示贵州吸毒人群 HCV 感染者的基因型主要为 3 型和 6 型、少数为 1 型，未发现 2 型、4 型和 5 型；性别和吸毒方式影响吸毒人群 HCV 感染者基因亚型的分布。以上结果可为当地合理制定该人群 HCV 治疗方案和估计公共卫生投入，提供有意义的参考。

参考文献

[1] Micallef JM, Kaldor JM, Dore GJ. Spontaneous viral clearance following acute hepatitis C infection: a systematic review of longitudinal studies [J]. J Viral Hepatitis, 2006, 13 (1): 34-41.

[2] Hoofnagle JH. Course and outcome of hepatitis C [J]. Hepatology, 2002, 36 (5): s21-s29.

[3] Thomas DL, Seeff LB. Natural history of hepatitis C [J]. Clin Liver Dis, 2005, 9 (3): 383-398.

[4] Di Bisceglle AM, Hoofnagle JH. Optimal therapy of hepatitis C [J]. Hepatology, 2002, 36 (5): s121-s127.

[5] Walsh MJ, Jonsson JR, Richardson MM. Non-response to antiviral therapy is associated with obesity and increased hepatic expression of SOC-3 in patients with chronic hepatitis C, viral gneotype 1 [J]. Gut, 2006, 55 (4): 529-535.

[6] Zeuzem S, Feinman SV, Rasenack J, *et al*. Peginterferon alfa-2a in patients with chronic hepatitis C [J]. N Engl J Med, 2000, 343 (23): 1666-1672.

[7] Lindsay KL, Trepo C, Heintges T, *et al*. A randomized, double-blind trial comparing pegylated interferon alfa-2b to interferon alfa-2b as initial treatment for chronic hepatitis C [J]. Hepatology, 2001, 34 (2): 395-403.

[8] Bowdent DS, Berzsenyi MD. Chronic hepatitis C virus infection: genotyping and its clinical role [J]. Future Microbiol, 2006, 1 (1): 103-112.

[9] Zeuzem S, Berg T, Moeller B, *et al*. Expert opinion on the treatment of patients with chronic hepatitis C [J]. J Viral Hepat, 2009, 16 (2): 75-90.

[10] Nguyen MH, Keeff EB. Prevalence and treatment of hepatitis C virus genotypes 4, 5 and 6 [J]. Clin Gastroenterol Hepatol, 2005, 3 Suppl 2: s97-s101.

[11] Dev AT, McCaw R, Sundararajan V, *et al*. South-East Asian patients with chronic hepatitis C: the impact of novel genotypes and race on treatment outcome [J]. Hepatology, 2002, 36 (5 II): 1259-1265.

[12] Ahmad W, Ijaz B, Javed FT, *et al*. HCV genotype-specific correlation with serum markers: higher predictability for genotype 4a [J]. Virol J, 2011, 8 (1): 293.

[13] Smith DB, Mellor J, Jarvis LM, *et al*. Variation of the hepatitis C virus 5′ non-conding region: implication for secondary struture, virus detection and typing. The International HCV Collaborative Study [J]. Gen Virol, 1995, 76 (pt 7): 1749-1761.

[14] Mellor J, Walsh MJ, Prescott LE, *et al*. Survey of type 6 group variants of hepatitis C virus in South-East Asia by using a core-based genotyping assay [J]. J Clin Microbiol, 1996, 34 (2): 417-423.

[15] Sandres-Saune K, Deny P, Pasquier C, *et al*. Determining hepatitis C genotype by analyzing the sequence of the NS5b region [J]. J Virol Meth, 2003, 109 (2): 187-193.

[16] Cantaloube JF, Laperche S, Gallian P, *et al*. Analysis of the 5′ noncoding region versus the NS5b region in genotyping hepatitis C virus isolates from blood donors in France [J]. J Clin Microbiol, 2006, 44 (6): 2051-2056.

[17] Laperche S, Lunel F, Izopet J, *et al*. Comparison of hepatitis C virus NS5b and 5′ noncoding gene sequencing methods in a multicenter study [J]. J Clin Microbiol, 2005, 43 (2): 733-739.

[18] Stelzl E, Van Der Meer C, Gouw R, *et al*. Determination of the hepatitis C virus subtype: comparison of sequencing and reverse hybridization assays [J]. Clin Chem Lab Med, 2007, 75 (pt 5): 1053-1061.

[19] Cochrane A, Searle B, Hardie A, *et al*. A genetic analysis of hepatitis C virus transmission between injection drug users [J]. J Infect Dis, 2002, 186 (9): 1212-1221.

[20] Zhang YY, Lok AS, Chan DT, *et al*. Greater diversity of hepatitis C virus genotypes found in Hong Kong than in mainland China [J]. J Clin Microbiol, 1995, 33 (11): 2931-2934.

[21] Simmonds P. Reconstructing the origins os human hepatitis viruses [J]. Philos Trans R Soc Lond B Biol Sci, 2001, 356 (1411): 1013-1026.

[22] Hu B, Zhang XW, Cheng G, *et al*. Genotyping of hepatitis C virus obtained from patients in Guangdong area [J]. Redai Yixue, 2006, 6 (10): 1071-1072. (in Chinese)
胡斌，张孝文，程钢，等. 广东地区丙型肝炎患者的 HCV **基因分型研究** [J]. **热带医学**, 2006, 6 (10): 1071-1072.

[23] Elasifer HA, Agnnyia YM, Al-Alagi BA, *et al*. Epidemiological manifestations of hepatitis C virus genotypes and its association with potential risk factors among Libyan patients [J]. Virol J, 2010, 7 (1): 317.

[24] Van Den Berg CHSB, Van De Laar TJW, Kok A, *et al*. Never injected, but hepatitis C virus-infected: a study among self-declared never-injecting drug users from the Amsterdam Cohort Studies [J]. J Viral Hepatitis, 2009, 16 (8): 568-577.

收稿日期: 2012-02-06 **修回日期**: 2012-02-17 **责任编辑**: 刘磊

© 1994-2012 China Academic Journal Electronic Publishing House. All rights reserved. http://www.cnki.net
